# Type 2 diabetic patients on renal replacement therapy: Probability to receive renal transplantation and survival after transplantation

**DOI:** 10.1371/journal.pone.0201478

**Published:** 2018-08-15

**Authors:** Marjo H. Kervinen, Seppo Lehto, Jaakko Helve, Carola Grönhagen-Riska, Patrik Finne

**Affiliations:** 1 Centre of Medicine, Kuopio University Hospital, Kuopio, Finland; 2 Varkaus Hospital, Varkaus, Finland; 3 Finnish Registry for Kidney Diseases, Helsinki, Finland; 4 Abdominal Center Nephrology, University of Helsinki and Helsinki University Hospital, Helsinki, Finland; Istituto Di Ricerche Farmacologiche Mario Negri, ITALY

## Abstract

**Background:**

Type 2 diabetic (T2DM) patients on renal replacement therapy (RRT) seldom receive a kidney transplant, which is partly due to age and comorbidities. Adjusting for case mix, we investigated whether T2DM patients have equal opportunity for renal transplantation compared to other patients on dialysis, and whether survival after transplantation is comparable.

**Methods:**

Patients who entered RRT in Finland in 2000–2010 (n = 5419) were identified from the Finnish Registry for Kidney Diseases and followed until the end of 2012. Of these, 20% had T2DM, 14% type 1 diabetes (T1DM) and 66% other than diabetes as the cause of ESRD. Uni-/multivariate survival analysis techniques were employed to assess the probability of kidney transplantation after the start of dialysis and survival after transplantation.

**Results:**

T2DM patients had a relative probability of renal transplantation of 0.18 (95% CI 0.15–0.22, P<0.001) compared to T1DM patients: this increased to 0.51 (95% CI 0.36–0.72, P<0.001) after adjustment for case mix (age, gender, laboratory values and comorbidities). When T2DM patients were compared to non-diabetic patients, the corresponding relative probabilities were 0.25 (95% CI 0.20–0.30, P<0.001) and 0.59 (95% CI 0.43–0.83, P = 0.002). After renal transplantation when adjusted for age and gender, relative risk of death was 1.25 (95% CI 0.64–2.44, P = 0.518) for T1DM patients and 0.72 (0.43–1.22, P = 0.227) for other patients compared to T2DM patients.

**Conclusions:**

T2DM patients had a considerably lower probability of receiving a kidney transplant, which could not be fully explained by differences in the patient characteristics. Survival within 5 years after transplantation is comparably good in T2DM patients.

## Introduction

With the increasing prevalence of type 2 diabetes worldwide, renal replacement therapy because of type 2 diabetes has also increased in the past decades. As the care of diabetes has improved, type 2 diabetic patients more often survive until RRT begins. Change in acceptance criteria for RRT has also contributed to this development, and type 2 diabetes is nowadays the most common diagnosis leading to RRT in Europe and the United States [1]. Survival of type 2 diabetic patients on RRT has improved since 1990–1995 [2].

According to earlier reports, type 2 diabetic patients on RRT do not easily access the wait list for transplantation. In a French study, patients older than 60 years had a very poor access to the wait list, regardless of diabetes status, and among 40-59-year-olds type 2 diabetes was connected to clearly decreased probability of waitlisting [3]. The age of type 2 diabetes patients who start RRT has shown to have increased over time [4]. We have earlier demonstrated that vascular comorbidities are related to decreased probability of receiving a kidney transplant among type 2 diabetes on dialysis [5]. In Finland 7% of all renal transplantations in 2015 were performed in patients with type 2 diabetes as cause of renal failure, and this proportion varied in the range of 4–12% in the countries of Sweden, Denmark, Norway, The Netherlands, Dutch-speaking and French-speaking Belgium (source: ERA-EDTA Registry, M. Pippias, 17 May 2018, personal communication). This proportion is small when compared to the proportion of type 2 diabetic patients of all patients who enter chronic dialysis treatment.

Our aim was to study factors related to the probability of type 2 diabetic patients to receive a kidney transplant and to compare this probability to patients with type 1 diabetes or other causes of end-stage renal disease (ESRD) on RRT. Many earlier reports consist of diabetic patients as a large group including both type 1 and type 2 diabetic patients. Our hypothesis was that RRT patients with type 2 diabetes have a lower probability of kidney transplantation per se even after considering confounding factors such as age and comorbidities. We also estimated whether the survival of type 2 diabetic patients with a kidney graft is comparable to that of patients with other diagnoses.

## Materials and methods

Patients who entered RRT in Finland in 2000–2010 were identified from the Finnish Registry for Kidney Diseases, and all these patients were included in the study. This registry is maintained by the Finnish Kidney and Liver Organization and is supported by Finnish governmental grants. The registry has been estimated to cover 97–99% of all RRT patients in Finland [6]. The registry contains information on the types of primary kidney disease and vascular comorbidities such as coronary heart disease, peripheral vascular disease, amputations and cerebrovascular disease at the beginning of RRT. Primary renal disease (e.g. ESRD due to type 1 or type 2 diabetes) was reported to the registry by the treating nephrologist as ICD-10 codes. In the registry coronary heart disease is defined by symptoms of it, earlier myocardial infarction, findings in the coronary angiography or by-pass operation or angioplasty of coronary arteries. Peripheral vascular disease is defined by symptoms of peripheral vascular disease, atherosclerotic findings in angiography of peripheral arteries or by-pass operation or angioplasty of peripheral arteries or earlier limb amputation. Information is also available on body mass index and laboratory values such as lipid profile, glycated haemoglobin A_1c_, serum C-reactive protein (CRP), albumin, phosphorus and calcium in the Registry. In Finland, all type 2 diabetes patients that are put on the wait list for kidney transplantation, are active candidates in the beginning at least. In the evaluation of the patient to be eligible for the kidney transplantation, patient's heart and arteries are examined, patient's cancer history, history of compliance for treatment and medication is evaluated and laboratory values and radiological findings are checked. The possibly underlying infectious diseases and co-morbidities are well treated before wait listing. The patient should try to be made as eligible for the transplantation as possible. Age itself is not seen as a limit, the overall condition and the evaluated benefits and risk for kidney operation count. The patients with type 2 diabetes as cause of RRT were compared with type 1 diabetic patients and non-diabetic patients starting RRT. Cumulative incidence of the first kidney transplantation was calculated using a method that takes death into account as a competing risk event [7]. Patients were taken to the study from start of RRT.

The Mann-Whitney U test for continuous variables and the chi-square test for categorical variables were used to compare the basic patient characteristics between groups.

Relative probabilities of kidney transplantation associated with baseline explanatory variables, e.g. age, gender, laboratory values and vascular co-morbidities, were estimated by fitting a proportional subdistribution hazards regression model that takes death into account as a competing risk event [8]. The R statistical software (version 3.0.3, The R Foundation for Statistical Computing, Vienna, Austria; available at http://www.r-project.org) with the 'cmprsk'-package was applied for estimating cumulative probability of kidney transplantation ('cuminc'-function) and for performing proportional subdistribution hazards regression ('crr'-function). In these analyses, kidney transplantation was the event of interest, death was analysed as a competing risk event and subjects were censored at the end of follow-up on December 31, 2012.

We furthermore studied patients who received a kidney graft. Survival of transplanted patients was studied using Kaplan-Meier curves and Cox proportional hazards regression. The patients were followed from the kidney transplantation until death or censoring on 31 December 2012.

The study has been conducted according to the principles of the Declaration of Helsinki. The study has been reviewed and approved by the Ethics Committee of Kuopio University Hospital. The data were received from the Finnish Registry for Kidney Diseases after permission and analysed anonymously.

## Results

A total of 5419 patients entered RRT in Finland in 2000–2010. The cause of ESRD was type 2 diabetes in 20%, type 1 diabetes in 14% and other in 66%. Table 1 shows the characteristics of the patients according to these three groups. At the start of RRT, type 2 diabetic patients had more comorbidities than patients with other causes of ESRD. Patients with type 2 diabetes were older, more obese and had lower serum levels of albumin and HDL cholesterol than other patients, and they had lower HbA_1c_ values than type 1 diabetic patients. Type 2 diabetic patients had peritoneal dialysis as the first dialysis mode less frequently than others. Characteristics at the start of RRT of patients who had received a kidney transplant are presented according to ESRD group in Table 2. Vascular comorbidities were more frequent in type 2 diabetic patients than in the two other ESRD groups, with the exception of peripheral vascular disease, which was as frequent among type 1 diabetic patients. 376 (49%) of type 1 diabetes patients, 105 (10%) of type 2 diabetes patients and 1267 (35%) other patients on RRT received a kidney transplant in the study period. Those type 1 diabetes patients, who received a kidney transplant, were 16% of the wait list time out of the wait list, type 2 diabetes patients 14% and non-diabetic patients 12%.

**Table 1:** Characteristics of type 1 and type 2 diabetes and non-diabetic patients starting RRT.

Variables in all patients	T1DM^a^	T2DM^b^	Non-DM^c^	P between T1DM and T2DM	P between groups T2DM and non-DM patients
**Patients who received a kidney transplant during follow-up/all patients in the group (%)**	376/768 (49%)	105/1065 (10%)	1267/3586 (35%)	<0.001	<0.001
**Age at start of RRT (years, mean)**	46.4±10.6	66.0±9.1	60.5±17.4	<0.001	<0.001
**Gender (M/F)**	491 (64%) / 277 (36%)	707 (66%) / 358 (34%)	2201 (63%) / 1280 (37%)	0.276	0.060
**HD**^**d**^**/PD**^**e**^	408 (53%) / 360 (47%)	886 (83%) / 179 (17%)	2672 (75%) / 801 (22%)	<0.001	<0.001
**Albumin (g/l)**	32±6	31±6	33±7	0.232	<0.001
**Ionized Calcium (mmol/l)**	1.16±0.11	1.14±0.12	1.16±0.13	0.011	<0.001
**Phosphorus (mmol/l)**	1.91±0.55	1.88±0.59	1.88±0.65	0.059	0.590
**Hemoglobin (g/l)**	110±16	107±15	107±16	<0.001	0.586
**CRP**^**f**^ **(g/l)**	15±33	21±38	26±48	<0.001	0.942
**Cholesterol (mmol/l)**	4.3±1.3	3.9±1.3	4.3±1.4	<0.001	<0.001
**HDL-cholesterol (mmol/l)**	1.3±0.5	1.1±0.4	1.2±0.5	<0.001	<0.001
**Triglycerides (mmol/l)**	1.6±1.0	1.8±1.7	1.7±1.2	0.024	0.147
**HbA**_**1c**_ **(% and mmol/l)**	8.4±1.9 and 68±	7.3±1.4 and 56±	5.9±0.9 and 41±	<0.001	<0.001
**SBP**^**g**^ **(mmHg)**	157±25	154±25	145±24	0.066	<0.001
**DBP**^**h**^ **(mmHg)**	84±13	78±13	81±14	<0.001	<0.001
**BMI (kg/m**^**2**^**) mean±SD and BMI median**	25±5 and 24	31±6 and 30	26±5 and 25	<0.001	<0.001
**Angina pectoris n (% of patients)**	110 (14)	342 (32)	535 (15)	<0.001	<0.001
**Previous myocardial infarction n (% of patients)**	117 (15)	234 (22)	408 (11)	<0.001	<0.001
**Previous coronary intervention n (% of patients)**	95 (12)	178 (17)	319 (9)	0.010	<0.001
**Decompensation of heart n (% of patients)**	51 (7)	194 (18)	278 (8)	<0.001	<0.001
**Atherosclerosis n (% of patients)**	123 (16)	265 (25)	246 (7)	<0.001	<0.001
**Previous limb amputation n (% of patients)**	80 (10)	116 (11)	27 (1)	0.745	<0.001
**Atherosclerotic surgery n (% of patients)**	59 (8)	133 (12)	168 (5)	0.001	<0.001
**Previous stroke n (% of patients)**	82 (11)	171 (16)	314 (9)	0.001	<0.001
**History of cancer (% of patients)**	3	9	15	0.515	0.061
**Blood pressure medication at start of RRT (% of patients)**	92	89	79	0.009	<0.001

^a^T1DM = diabetes mellitus type 1

^b^T2DM = diabetes mellitus type 2

^c^Non-DM = other cause of end-stage renal disease than diabetes

^d^HD, hemodialysis

^e^PD, peritoneal dialysis

^f^CRP, C-reactive protein

^g^SBP, systolic blood pressure

^h^DBP, diastolic blood pressure

Numbers are means ± SD.

**Table 2:** Characteristics of type 1 and type 2 diabetes and non-diabetic patients who received first renal transplantation during study period in Finland.

Variables in patients who received a kidney transplant	T1DM^a^ (n = 376)	T2DM^b^ (n = 105)	Non-DM^c^ (n = 1267)	P between T1DM and T2DM patients	P between T2DM and non-DM patients
**Age at start of RRT (years, mean)**	42.8	57.3	46.8	<0.001	<0.001
**Average dialysis time before transplantation (years)**	2.0±1.4	2.4±1.5	1.9±1.5	<0.001	<0.001
**Mean age at time of first kidney transplantation (years)**	44.8±9.0	59.7±8.4	48.8±17.3	<0.001	<0.001
**Gender M/F**	246 (65%) / 130 (35%)	89 (85%) / 16 (15%)	782 (62%) / 485 (38%)	<0.001	<0.001
**HD**^**d**^**/PD**^**e**^	167/209	69/36	811/448	<0.001	0.691
**Albumin (g/l)**	33±6	32±6	35±7	0.850	<0.001
**Ionized Calcium (mmol/l)**	1.16±0.11	1.14±0.12	1.18±0.13	0.118	0.002
**Phosphorus (mmol/l)**	1.92±0.55	1.88±0.49	1.91±0.61	0.736	0.754
**Hemoglobin (g/l)**	112±16	112±13	110±16	0.489	0.942
**CRP**^**f**^ **(g/l)**	8±16	10±11	13±29	0.081	0.616
**Cholesterol (mmol/l)**	4.4±1.1	4.0±1.5	4.5±1.4	0.001	<0.001
**HDL-cholesterol (mmol/l)**	1.4±0.5	1.2±0.6	1.3±0.5	<0.001	0.006
**Triglycerides (mmol/l)**	1.6±0.9	1.8±1.0	1.8±1.5	0.414	0.951
**HbA1c (% and mmol/l)**	8.4±2.0 and 68±	7.1±1.4 and 54±	5.6±0.8 and 38±	<0.001	<0.001
**SBP**^**g**^ **(mmHg)**	156±24	159±24	145±20	0.269	<0.001
**DBP**^**h**^ **(mmHg)**	86±13	83±12	86±12	0.019	0.090
**BMI (kg/m**^**2**^**) mean±SD and BMI median**	25±4 and 24	29±5 and 28	25±5 and 25	<0.001	<0.001
**Angina pectoris (% of patients)**	5	10	2	0.064	<0.001
**Previous myocardial infarction (% of patients)**	6	8	2	0.626	0.001
**Previous coronary intervention (% of patients)**	7	7	3	0.885	0.062
**Decompensation of heart (% of patients)**	1	4	1	0.100	0.008
**Atherosclerosis (% of patients)**	5	1	1	0.081	0.829
**Previous limb amputation (% of patients)**	2	0	0	0.115	0.624
**Atherosclerotic surgery (% of patients)**	1	1	1	0.777	0.863
**Previous stroke (% of patients)**	6	10	4	0.082	0.001
**History of cancer (% of patients)**	2	3	4	0.490	0.797

^a^T1DM = diabetes mellitus type 1

^b^T2DM = diabetes mellitus type 2

^c^Non-DM = other cause of end-stage renal disease than diabetes

^d^HD, hemodialysis

^e^PD, peritoneal dialysis

^f^CRP, C-reactive protein

^g^SBP, systolic blood pressure

^h^DBP, diastolic blood pressure

Numbers are means ± SD.

Type 2 diabetic patients less frequently received a kidney transplant than type 1 diabetic patients. There were no type 2 diabetic patients with simultaneous pancreas-kidney transplantations in the study group. During the study period, we only had 11 simultaneous pancreas-kidney transplantations in type 1 diabetes patients.

Of type 2 diabetic patients on RRT, only 105 (10%) had received a kidney transplant by the end of study period (Table 1), and females more seldom than males (4% vs. 13%). The transplants of type 2 diabetic patients were all except one from deceased donors. In comparison, 13 (2%) of the type 1 diabetic patients received kidney transplants from living donors and 363 (47%) from deceased donors.

When compared with type 1 diabetic patients, patients with type 2 diabetes had a relative probability of renal transplantation of 0.18 (95% CI 0.15–0.22, P<0.001), and when adjusted for age and gender it was 0.41 (95%CI 0.33–0.51, P<0.001). With further adjustment for laboratory variables and for comorbidities (angina pectoris, myocardial infarctions, coronary interventions, peripheral vascular disease, limb amputations and strokes) relative probability was 0.51 (95% CI 0.36–0.72, P<0.001).

When type 2 diabetic patients were compared with non-diabetic patients, the relative probability of renal transplantation was 0.25 (95% CI 0.20–0.30, P<0.001) and with adjustment for age and gender it was 0.35 (95% CI 0.28–0.42, P<0.001). When further adjusting for laboratory values and comorbidities the relative probability was 0.59 (95% CI 0.43–0.83, P = 0.002).

**Fig 1:**
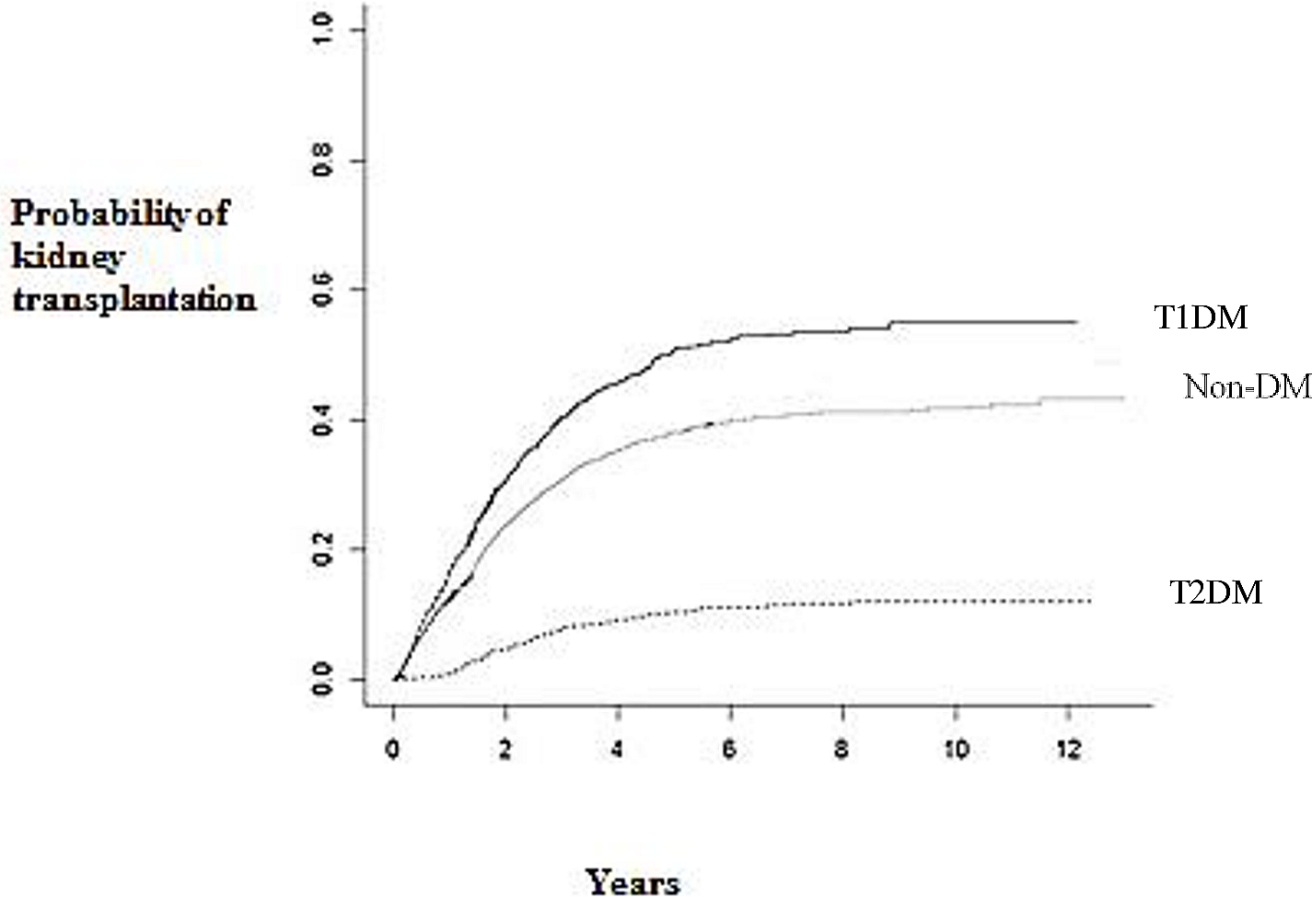
Cumulative incidence of renal transplantation in type 1 and type 2 diabetes and non-diabetic patients. Cumulative incidence of renal transplantation in type 1 (straight black line), type 2 diabetic patients (dotted line) and in patients without diabetes (straight grey line) on RRT in Finland in 2000–2010 from start of renal replacement therapy. T1DM = type 1 diabetic patients, T2DM = type 2 diabetic patients, Non-DM = non-diabetic patients.

The cumulative incidence of renal transplantation in the three ESRD groups is presented in Fig 1.

In Kaplan-Meier curves the median calculated time from start of RRT to renal transplantation was 3.3 (95% CI 2.8–3.7) years for patients with type 1 diabetes while median time could not be calculated for type 2 diabetic patients because 50% probability of transplantation was not reached. All non-diabetic patients had a median calculated time for renal transplantation of 5.1 years (95% CI 4.6–5.7). The probability to get a kidney transplant within 5 years from start of RRT was 17% among type 2 diabetic patients, 64% among type 1 diabetic patients and 50% among non-diabetic patients.

**Fig 2:**
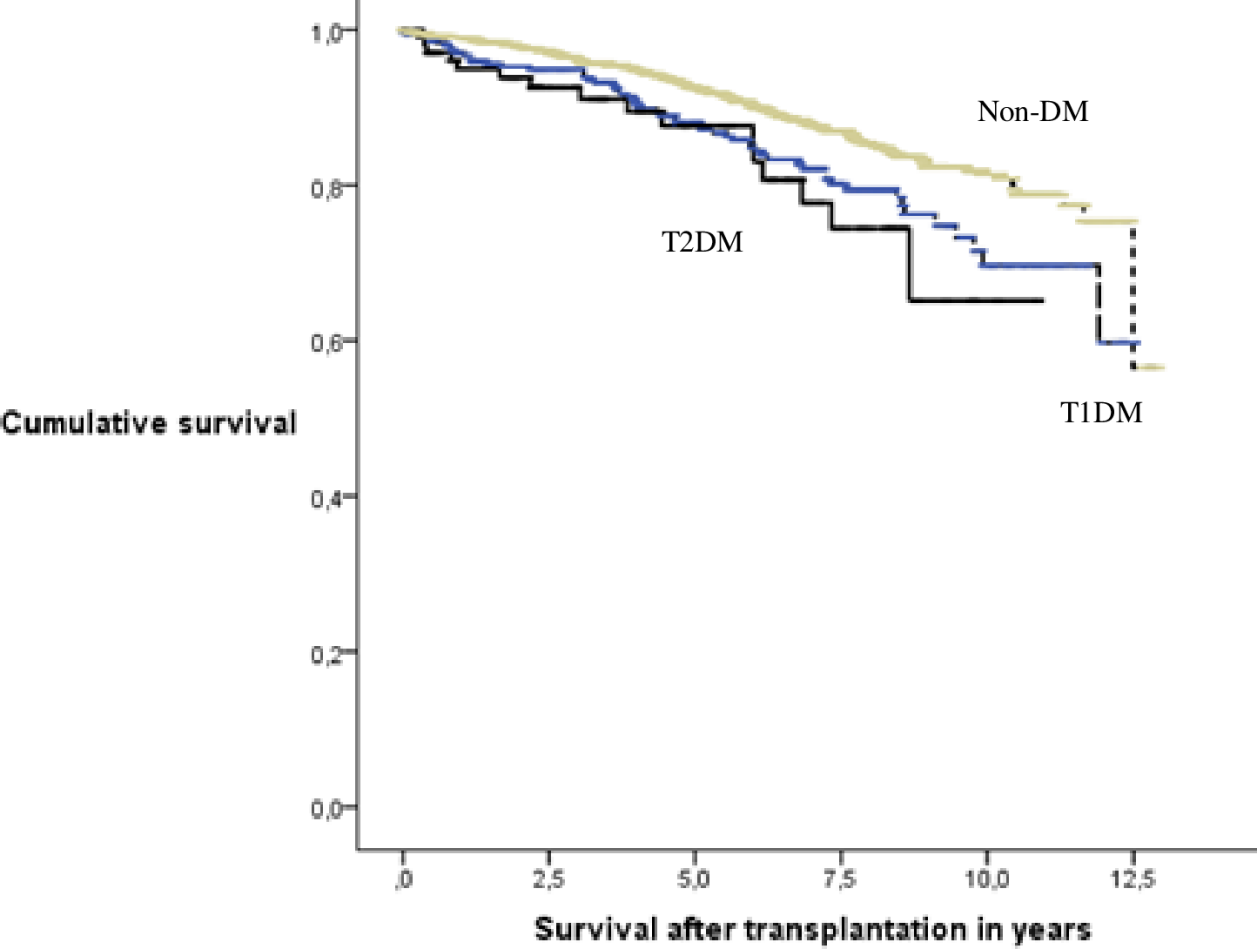
Cumulative survival probability after renal transplantation in type 1 and type 2 diabetes and non-diabetic patients. Cumulative survival probability in years after renal transplantation in type1 (––line), type 2 (—line) diabetic patients and in patients without diabetes (—line) in Finland in 2000–2010. T1DM = type 1 diabetic patients, T2DM = type 2 diabetic patients, Non-DM = non-diabetic patients.

Crude survival probabilities after transplantation are presented in Fig 2. At five years after transplantation, survival probability of type 2 diabetic patients was 88%, of type 1 diabetic patients 88% and of non-diabetic patients 93%. The causes of death in patients who received a kidney transplant was most often cardiovascular: 44% of the deceased type 2 diabetic patients, 60% of the deceased type 1 diabetic patients and 37% of non-diabetic deceased study patients. When transplant recipients with type 2 diabetes were compared to those with type 1 diabetes, age- and gender adjusted HR for death was 0.80 (95% CI 0.41–1.57, P = 0.518), and when compared to those without diabetes, it was 1.39 (95% CI 0.82–2.35, P = 0.227).

There was no difference in the wait list time to transplantation between transplanted patients with type 2 and type 1 diabetes (P = 0.373), but type 2 diabetic patients had longer wait list time than patients without diabetes (P = 0.025). Type 2 diabetes patients seem to get access to the wait list more slowly than type 1 diabetes patients, since (in Table 2) the average dialysis time before transplantation is longer with type 2 diabetes patients than with type 1 diabetes patients. The average wait list time for kidney transplantation in Finland is approximately 2 years. It must be noted that we studied the wait list times only for those who received a kidney transplant, not for those who were withdrawn from the list or died while on the wait list. We examined how many days the patients who received the first kidney transplant were active on the list and how many days they were temporarily out of the wait list. For type 1 diabetes patients, who received a kidney transplant, they were out of the wait list 16% of the waitlist time, for type 2 diabetes 14% and for others 12% (p = 0.021). It must be noted, that in Finland patients over BMI 32–34 have not generally been earlier accepted to the wait list before reducing wait because of more complications seen on overweight patients at transplantation but nowadays this limit is more flexible.

## Discussion

Our study shows that type 2 diabetic patients on RRT have a lower probability of receiving kidney transplantation than type 1 diabetic patients or patients without diabetes on dialysis. The difference is partly, but not totally, explained by differences between the patient groups. Age and vascular comorbidities contribute to the reduced probability of type 2 diabetic patients to get on the wait list for transplantation, but there appear to be other and partly unknown factors as well. Within 5 years after transplantation, the survival of type 2 diabetic patients was as good as that of patients with other causes of end-stage renal disease. It should be noted that certain variables regarding the outcome of transplant, like blood group and panel reactive antibodies were not available.

The focus of this study was to investigate all ESRD patients and their possibility of getting a kidney transplant. As stated out there was not difference in the wait list time between type 1 and type 2 diabetes patients.

We observed a tendency that female type 2 diabetic patients received a renal transplantation less often than male patients. The reason for this is not known. Such a sex difference was not observed among type 1 diabetic patients or patients without diabetes, and these results need to be confirmed in a larger population. Sex differences according to level of body mass index have been reported earlier in overall dialysis patients receiving renal transplantation [9]. Also, access to transplantation in women with comorbidities was lower than that of males in a study by Segev et al, but in their study type 1 and type 2 diabetes were not studied separately [10]. Psychosocial factors that are not collected in the Registry may have effect on the sex difference. In Finland, use of kidneys from living donors has been rare. In type 2 diabetic patients, the older age of the patients may affect the possibility of potential living donors. In our study the type 2 diabetic patients had a longer wait list time for transplantation than patients without diabetes. One reason for this apart from age may be the fact that diabetic patients were temporarily off the waitlist because of infections and other complications [11]. In the study by Kyllönen and Salmela Finnish diabetes patients on the wait list had no significant different mean active wait list times when looking at the transplantated patients and those still on the active list, but for patients who had died or were withdrawn from the wait list, the inactive time exceeded 50% in 1993–2000. In their study 11% of diabetes patients on the wait list had died and 3% had been permanently withdrawn from the wait list. The low proportion of patients with type 2 diabetes as cause of renal failure among all patients who received a kidney transplantation is not unique for Finland, but similar in several other countries in Northern Europe (source: ERA-EDTA Registry, M. Pippias, 17 May 2018, personal communication).

The older age of type 2 diabetic patients on RRT is certainly one reason for not getting a transplantation, though there is no specific age limit for transplantation. Elderly transplantation patients have been shown to have lower risk of death compared to waitlisted dialysis patients of same age [12,13]. Also, diabetic patients over 70 years of age had a clear benefit from kidney transplantation in the study by Rao et al [12]. Our patients are similarly often elderly or near that in Finnish type 2 diabetes patients on RRT. Also living donor transplantations benefit the elderly kidney patients, but in type 2 diabetes patients they are rare, and other options like accepting the expanded criteria donor organs should be considered [13]. Our study emphasizes the need for further investigation of the reasons why type 2 diabetic patients have a poorer probability of getting a renal transplantation. The observed factors probably do not explain the current situation in whole. In our study, BMI was clearly associated with the probability of kidney transplantation in type 2 diabetic patients. Some patients may never reach the wait list because of high body weight [14]. In the study by Huang et al [14], in patients with weight inappropriate for transplantation, diabetic ESRD was negatively associated with conversion to active wait list status. Therefore, methods to reach reasonable BMI levels should be considered. In Finland, the acceptable BMI has earlier been lower than 32–34 kg/m^2^, but in many other countries patients with higher BMI have been accepted for transplantation. As type 2 diabetes patients are older and heavier, start less their RRT as PD than type 1 diabetes patients and have more often co-morbidities than type 1 diabetes patients, it may be, that type 2 diabetes patients are probably less seen as potential kidney transplantation patients and less in the evaluation for kidney transplantation at all.

Survival of type 2 diabetic patients with a kidney transplant, although fairly good in this selected group, was somewhat but not significantly lower than survival of patients without diabetes. In a study by Kute VB et al [15], the 4-year patient survival after kidney transplantation with organs from deceased donors in type 2 diabetes was 54%, but the results were better with living donors. Rocha et al. showed a fairly poor survival (69% at 5 years and 50% at 10 years) of diabetic kidney transplant recipients [16]. In a study by Cosio et al. [17] including both deceased donor and living kidney donor transplantations, diabetic (all types of diabetes) patients had reduced 5-year survival (70% vs. 93%) compared to patients without diabetes, but the 5-year mortality of patients with diabetes has declined significantly over time [18]. In our study, the survival of type 2 diabetic patients after transplantation was good (12% risk of death at 5 years from transplantation) even though almost all kidney grafts came from deceased donors. On the other hand, the fairly good prognosis may indicate that selection of type 2 diabetic patients for transplantation is too strict, and a larger number of patients could possibly benefit from transplantation.

There are a few limitations in our study. First, the amount of type 2 diabetes patients receiving kidney transplantation was relatively small, 105, and almost all received the new kidney from deceased donors. Second, we do not have pre-emptive transplantations for type 2 diabetes patients in the group. It could give an even better survival for the group after transplantation. Third, the transplantation criteria for type 2 diabetes patients may be somewhat tight in Finland, which may, on the other hand, give a better survival for the type 2 diabetes patients in the study. We see the lack of information on referral practices of type 2 diabetes patients with ESRD as a limitation of our study. And fourth, the variables for good prognosis in kidney transplantation would need more information on the operation itself, HLA-match, etc. There may also be other earlier unknown factors as well, like psychosocial factors, that are not collected in the Registry.

Nowadays there are different options for transplantation in type 2 diabetes [19]. Some countries have performed pre-emptive transplantations with deceased donorsˈ or living related donorsˈ organs to type 2 diabetic patients. A limitation for living donors to type 2 diabetes patients can be the age of the patients and the lack of possible donors in the family or spouses. The ideal timing for renal transplantation would be prior to start of dialysis or as soon as possible after dialysis has been initiated as a prolonged dialysis time has been associated to impaired survival [20]. As type 2 diabetic patients are older than other patients on dialysis a long waiting time might reduce their possibility to ever get a transplant. There has also been some concern about the relatively fast recurrence of diabetic nephropathy in some type 2 diabetic patients after transplantation [21], but this is not very common in our experience. Such findings might partly lead to hesitation to waitlist elderly and type 2 diabetic patients for renal transplantation. Kidney transplantations are nowadays quite commonly performed among patients older than 70 years.

It should be noted that primary kidney diagnosis in our study was not confirmed with a kidney biopsy. Kidney biopsies are rare in this patient group, also in other countries, as the diagnosis often is clear by clinical evaluation, e.g. when other diabetes complications such as retinopathy are present. However, some of the patients might have had other causes for renal failure. In a study by Bell et al [22], the primary kidney diagnosis of patients with type 2 diabetes displayed more heterogeneity than in patients with type 1 diabetes, and a primary kidney diagnosis other than diabetic nephropathy did affect the survival of these patient groups.

Kidney transplantation seems to halt the progression of cardiovascular diseases, and survival of grafts is good in type 2 diabetes as well [23,24]. In another study by Kianda et al [25], adequate control of cardiovascular risk factors and early referral to transplantation might improve the eligibility for kidney transplantation in all patients. On the other hand, several studies have emphasized that the higher risk of cardiovascular events in diabetic patients persists despite transplantation [18].

Future clinical challenges in improving survival of type 2 diabetic patients with ESRD are how to keep these patients in good condition so that the vascular diseases, high body weight, or infections would not be a barrier for kidney transplantation, and how to best manage diabetes and preserve kidney function after kidney transplantation. Our study points out the need for more active evaluation for the possibilities of kidney transplantation in type 2 diabetic patients at least in Finland. In Australia and New Zealand, kidney transplant recipients with type 2 diabetes had substantially poorer patient survival than non-diabetic recipients [26]. The risk of death was especially increased with patients under 40 years in their study, and there was no evidence of improvement in mortality over time among patients with type 2 diabetes and a kidney graft. In our study, the mean age of type 2 diabetes patients at start of dialysis was 66 years and at time of first transplantation nearly 60 years. The right timing for this evaluation for transplantation early enough is also essential especially because emergency start of dialysis is known to worsen survival and, as in our study, these patients are older candidates for transplantation in general. Also, in France the incidence rate of RRT has shown to increase in 2005–2014, and especially in patients with type 2 diabetes as cause of ESRD, and improvement has been achieved with kidney transplantations and survival despite patient aging and heavy co-morbidities [27]. In Finland, where the amount of the whole population is not rising, the incidence of RRT due to type 2 diabetes after rising for years has shown to stabilize in the late years after year 2000, but in the very latest years (2016) the incidence has shown a small rise again. Although type 2 diabetic patients relatively seldom receive a renal transplant, their survival within 5 years after transplantation is comparable to patients with other causes of ESRD in Finland. Attention must be paid to these patients' many points of treatment after transplantation as well. Our finding should encourage attempts to increase renal transplantations in type 2 diabetic patients on RRT in this setting, but further studies are needed to find out more reasons for low referral to kidney transplantation in this patient group and to see which of these patients truly benefit from transplantation in long-term with regard of survival and quality of life.

## References

[1] Collins AJ, Foley RN, Herzog C, Chavers B, Gilbertson D, Herzog C, et al US Renal Data System 2012 Annual Data Report. Am J Kidney Dis. 2013 1;61(1 Suppl 1):A7, e1–476. 10.1053/j.ajkd.2012.11.031 23253259

[2] Kervinen M, Lehto S, Ikäheimo R, Karhapää P, Grönhagen-Riska C, Finne P. Improved survival of type 2 diabetic patients on renal replacement therapy in Finland. Nephrol Dial Transplant. 2010 3;25(3):892–6. 10.1093/ndt/gfp555 19846391

[3] Hourmant M, de Cornelissen F, Brunet P, Pavaday K, Assogba F, Couchoud C, et al; registre du REIN. Access to the waiting list and renal transplantation. Nephrol Ther. 2013 9;9 Suppl 1:S139–66. 24119579 10.1016/S1769-7255(13)70043-9

[4] Prischl FC, Auinger M, Säemann M, Mayer G, Rosenkranz AR, Wallner M, et al for the Austrian Dialysis and Transplant Registry. Diabetes-related end-stage renal disease in Austria 1965–2013. Nephrol Dial Transplant. 2015 11;30(11):1920–1927. 10.1093/ndt/gfv113 25977308

[5] Kervinen M, Lehto S, Grönhagen-Riska C, Finne P. Effect of vascular comorbidities on survival of type 2 diabetes patients on renal replacement therapy. Am J Nephrol. 2012;36(6):509–15. 10.1159/000345143 23171532

[6] Finnish Registry for Kidney diseases. Annual Report 2012. Helsinki, Finland. Finnish Registry for Kidney Diseases, 2013. Available from http://www.musili.fi/smtr/English.

[7] Kalbfleisch JD, Prentice RL. The Statistical Analysis of Failure Time Data; New York, NY, John Wiley, 1980.

[8] Fine JP, Gray RJ. A proportional hazards model for the subdistribution of a competing risk. J Am Stat Assoc. 1999; 94:496–509.

[9] Gill JS, Hendren E, Dong J, Johnston O, Gill J. Differential association of body mass index with access to kidney transplantation in men and women. Clin J Am Soc Nephrol. 2014 5;9(5):951–9. 10.2215/CJN.08310813 24742478 PMC4011447

[10] Segev DL, Kucirka LM, Oberai PC, Parekh RS, Boulware LE, Powe NR, et al Age and Comorbidities Are Effect Modifiers of Gender Disparities in Renal Transplantation. J Am Soc Nephrol. 2009 3;20(3):621–8. 10.1681/ASN.2008060591 19129311 PMC2653677

[11] Kyllönen L, Salmela K. Why do some diabetic patients on the kidney transplant waiting list not receive a transplant? Transpl Int. 2004 10;17(9):511–7. 10.1007/s00147-004-0754-z 15338122

[12] Rao PS, Merion RM, Ashby VB, Port FK, Wolfe RA, Kayler LK. Renal transplantation in elderly patients older than 70 years of age: results from the Scientific Registry of Transplant Recipients. Transplantation. 2007 4 27;83(8):1069–74. 10.1097/01.tp.0000259621.56861.31 17452897

[13] Knoll GA. Kidney transplantation in the older adult. Am J Kidney Dis. 2013 5;61(5):790–7. 10.1053/j.ajkd.2012.08.049 23261121

[14] Huang E, Shye M, Elashoff D, Mehrnia A, Bunnapradist S. Incidence of conversion to active waitlist status among temporarily inactive obese renal transplant candidates. Transplantation. 2014 7 27;98(2):177–86. 10.1097/TP.0000000000000037 24608735

[15] Kute VB, Vanikar AV, Trivedi HL, Shah PR, Goplani KR, Gumber MR, et al Outcome of renal transplantation in patients with diabetic nephropathy—a single-center experience. Int Urol Nephrol. 2011 6;43(2):535–41. 10.1007/s11255-010-9852-2 21107691

[16] Rocha A, Malheiro J, Martins LS, Fonseca I, Dias L, Pedroso S, et al Kidney transplantation in type 2 diabetic patients: a matched survival analysis. Transplant Proc. 2013 Jul-Aug;45(6):2141–6. 10.1016/j.transproceed.2012.11.013 23747181

[17] Cosio FG, Hickson LJ, Griffin MD, Stegall MD, Kudva Y. Patient survival and cardiovascular risk after kidney transplantation: the challenge of diabetes. Am J Transplant. 2008 3;8(3):593–9. 10.1111/j.1600-6143.2007.02101.x 18294155

[18] Keddis MT, El Ters M, Rodrigo E, Dean P, Wohlfahrtova M, Kudva YC, et al Enhanced posttransplant management of patients with diabetes improves patient outcomes. Kidney Int. 2014 9;86(3):610–8. 10.1038/ki.2014.70 24694990

[19] Fourtounas C. Transplant options for patients with type 2 diabetes and chronic kidney disease. World J Transplant. 2014 6 24;4(2):102–10. 10.5500/wjt.v4.i2.102 25032099 PMC4094945

[20] Helanterä I, Salmela K, Kyllönen L, Koskinen P, Grönhagen-Riska C, Finne P. Pretransplant dialysis duration and risk of death after kidney transplantation in the current era. Transplantation. 2014 8 27;98(4):458–64. 10.1097/TP.0000000000000085 24646770

[21] Tinel C, Martin L, Cabanne JF, Tanter Y, Rifle G, Mousson C. Early recurrence of Type 2 diabetic nephropathy after kidney transplantation. Clin Nephrol. 2011 2;75(2):179–80. 21255550

[22] Bell S, Fletcher EH, Brady I, Looker HC, Levin D, Joss N, et al; Scottish Diabetes Research Network and Scottish Renal Registry. End-stage renal disease and survival in people with diabetes: a national database linkage study. QJM. 2015 2;108(2):127–34. 10.1093/qjmed/hcu170 25140030 PMC4309927

[23] Luan FL, Samaniego M. Transplantation in diabetic kidney failure patients: modalities, outcomes, and clinical management. Semin Dial. 2010 Mar-Apr;23(2):198–205. 10.1111/j.1525-139X.2010.00708.x 20374550

[24] Guerra G, Ilahe A, Ciancio G. Diabetes and kidney transplantation: past, present, and future. Curr Diab Rep. 2012 10;12(5):597–603. 10.1007/s11892-012-0306-3 22872422

[25] Kianda MN, Wissing KM, Broeders NE, Lemy A, Ghisdal L, Hoang AD, et al Ineligibility for renal transplantation: prevalence, causes and survival in a consecutive cohort of 445 patients. Clin Transplant. 2011 Jul-Aug;25(4):576–83. 10.1111/j.1399-0012.2010.01317.x 20718825

[26] Lim WH, Wong G, Pilmore HL, McDonald SP, Chadban SJ. Long-term outcomes of kidney transplantation in people with type 2 diabetes: a population cohort study. Lancet Diabetes Endocrinol. 2017 1;5(1):26–33. 10.1016/S2213-8587(16)30317-5 28010785

[27] Vigneau C, Kolko A, Stengel B, Jacquelinet C, Landais P, Rieu P, et al; REIN registry. Ten-years trends in renal replacement therapy for end-stage renal disease in mainland France: Lessons from the French Renal Epidemiology and Information Network (REIN) registry. Nephrol Ther. 2017 2 1. pii: S1769-7255(16)30636-8. 10.1016/j.nephro.2016.07.453 28161264

